# Determinants of Adherence to the Mediterranean Diet in Spanish Children and Adolescents: The PASOS Study

**DOI:** 10.3390/nu14040738

**Published:** 2022-02-09

**Authors:** Maria del Mar Bibiloni, Laura Gallardo-Alfaro, Santiago F. Gómez, Julia Wärnberg, Maddi Osés-Recalde, Marcela González-Gross, Narcís Gusi, Susana Aznar, Elena Marín-Cascales, Miguel A. González-Valeiro, Lluís Serra-Majem, Nicolás Terrados, Marta Segu, Camille Lassale, Clara Homs, Juan Carlos Benavente-Marín, Idoia Labayen, Augusto G. Zapico, Jesús Sánchez-Gómez, Fabio Jiménez-Zazo, Pedro E. Alcaraz, Marta Sevilla-Sánchez, Estefanía Herrera-Ramos, Susana Pulgar, Clara Sistac, Helmut Schröder, Cristina Bouzas, Josep A. Tur

**Affiliations:** 1 Research Group on Community Nutrition and Oxidative Stress, University of Balearic Islands-IUNICS & Health Research Institute of the Balearic Islands (IDISBA), 07122 Palma de Mallorca, Spain; mar.bibiloni@uib.es (M.d.M.B.); lauragala3@gmail.com (L.G.-A.); cristina.bouzas@uib.es (C.B.); 2 Centro de Investigación Biomédica en Red Fisiopatología de la Obesidad y la Nutrición (CIBEROBN), Institute of Health Carlos III, 28029 Madrid, Spain; jwarnberg@uma.es (J.W.); marcela.gonzalez.gross@upm.es (M.G.-G.); lluis.serra@ulpgc.es (L.S.-M.); 3 Gasol Foundation, Sant Boi de Llobregat, 08830 Barcelona, Spain; sgomez@gasolfoundation.org (S.F.G.); choms@gasolfoundation.org (C.H.); 4 GREpS, Health Education Research Group, Nursing and Physiotherapy Department, University of Lleida, 25008 Lleida, Spain; 5 Epi-Phaan Research Group, Institute of Biomedical research of Malaga (IBIMA), Universidad de Málaga, 29016 Málaga, Spain; jc.benaventemarin@gmail.com; 6 ELIKOS Group, Department of Health Sciences, Institute for Innovation and Sustainable Development in Food Chain (IS-FOOD), Public University of Navarre, 31008 Pamplona, Spain; maddiosre@gmail.com (M.O.-R.); idoia.labayen@unavarra.es (I.L.); 7 ImFINE Research Group, Department of Health and Human Performance, Universidad Politécnica de Madrid, 28040 Madrid, Spain; agarciaz@ucm.es; 8 Physical Activity and Quality of Life Research Group (AFYCAV), Faculty of Sport Sciences, University of Extremadura, 10003 Cáceres, Spain; ngusi@unex.es (N.G.); jesanchezg@unex.es (J.S.-G.); 9 PAFS Research Group, Faculty of Sports Sciences, La Mancha-Toledo Campus, University of Castilla, 45071 Toledo, Spain; susana.aznar@uclm.es (S.A.); fabio_avila@hotmail.es (F.J.-Z.); 10 Research Center for High Performance Sport, San Antonio Catholic University of Murcia, 30107 Murcia, Spain; emarin@ucam.edu (E.M.-C.); palcaraz@ucam.edu (P.E.A.); 11 Faculty of Sport Sciences, San Antonio Catholic University of Murcia, 30107 Murcia, Spain; 12 Faculty of Sports Sciences and Physical Education, Universidade da Coruña, 15001 A Coruña, Spain; miguel.gonzalez.valeiro@udc.es (M.A.G.-V.); marta.sevilla@udc.es (M.S.-S.); 13 Research Institute of Biomedical and Health Sciences (IUIBS), University of Las Palmas de Gran Canaria, 35016 Las Palmas, Spain; faniya1@gmail.com; 14 Preventive Medicine Service, Centro Hospitalario Universitario Insular Materno Infantil (CHUIMI), Canarian Health Service, 35016 Las Palmas, Spain; 15 Regional Unit of Sports Medicine of Principado de Asturias, Municipal Sports Foundation of Avilés, 33401 Avilés, Spain; nterrados@aviles.es (N.T.); susanapulgarmunoz@gmail.com (S.P.); 16 Probitas Foundation, 08022 Barcelona, Spain; marta.segu@fundacionprobitas.org (M.S.); clara.sistac@fundacionprobitas.org (C.S.); 17 CIBER de Epidemiología y Salud Pública (CIBERESP), Instituto de Salud Carlos III, 28049 Madrid, Spain; classale@imim.es (C.L.); hschroeder@imim.es (H.S.); 18 Cardiovascular Risk and Nutrition Research Group, Hospital del Mar Institute for Medical Research, 08003 Barcelona, Spain; 19 Global Research on Wellbeing (GRoW), Blanquerna Faculty of Health Sciences, Ramon Llull University, 08022 Barcelona, Spain; 20 Department of Didactics of Language, Arts and Physical Education, Universidad Complutense de Madrid, 28040 Madrid, Spain

**Keywords:** children, adolescents, Mediterranean diet, lifestyle, PASOS

## Abstract

A progressive shift away from traditional healthy dietary patterns, such as the Mediterranean diet (MedDiet), has been observed in recent decades. The aim of this study was to assess determinants of optimal adherence to the MedDiet in Spanish children and adolescents. A cross-sectional analysis was included in the PASOS nationwide representative study in Spain. Participants were 3607 children and adolescents; 8–16 years old. Primary and secondary outcome measures of weight and height were measured. Adherence to the MedDiet, physical activity, and sedentary behavior in children and adolescents, as well as parental physical activity and dietary habits, were assessed. Optimal adherence to the MedDiet was observed in 45.5% of primary school students and 34.8% of secondary school students (OR: 0.65; 95%CI: 0.56–0.75). Optimal adherence to the MedDiet was higher in children/adolescents meeting daily recommended moderate and vigorous physical activity (OR: 2.39, 95%CI: 1.97–2.89) and in those meeting daily recommended screen time on weekdays (OR: 2.05, 95%CI: 1.77–2.38) and weekends (OR: 1.76, 95%CI: 1.48–2.10). Participants with optimal adherence to the MedDiet were more likely to have mothers with a high-level education and high-tercile of SDQS, mothers who never smoked or were former smokers, and mothers who met the recommended physical activity and screen time. It can be concluded that a low prevalence of optimal adherence to the MedDiet was found among current Spanish children and adolescents. Optimal adherence to the MedDiet was associated with reaching the recommendations on physical activity and screen time, with the highest maternal educational level, and healthier maternal lifestyles.

## 1. Introduction

High adherence to Mediterranean diet (MedDiet) in children and adolescents was associated with a healthier metabolic profile [1], academic performance [2], motivation and learning strategies [3], health-related quality of life [4,5], and satisfaction with life [6]. Despite the benefits of MedDiet, in Mediterranean countries, children and adolescents are deviating toward a ‘Western diet’ richer in saturated fat, refined grains, simple carbohydrates, and ultra-processed foods [7].

The KIDMED (Mediterranean Diet Quality Index for children and adolescents) score has been widely used to evaluate adherence to MedDiet in childhood [8] and showed that less than half of the Spanish child and adolescent population had a high adherence to MedDiet [2,6,9,10,11,12,13,14], which worsened with age in Spain [6,10,11,12,13].

Adherence to MedDiet in childhood is highly influenced by individual factors and parent’s socioeconomic status and lifestyle, including low physical activity and sedentary activities. The number of studies exploring these topics in Spanish childhood has increased [6,10,11,12,13,14]. Despite of the direct association between adherence to MedDiet and physical activity, and inverse association with sedentary behavior, the results for sex, age, socioeconomic status, and weight status are still inconsistent [15].

The aim of this study was to assess the determinants of optimal adherence to MedDiet in Spanish children and adolescents.

## 2. Methods

### 2.1. Study Design

Cross-sectional analysis within the Physical Activity, Sedentarism and Obesity in Spanish Youth (PASOS) study, an observational, nationally representative, and multicenter research work. Details of the PASOS study protocol are fully described in [16].

### 2.2. Participants, Recruitment, and Ethics

Participants were 8–16-year-old children and adolescents (n = 3607), recruited from March 2019 to February 2020 in 242 primary and secondary schools in 121 localities from all 17 of the Spanish autonomous communities. Individuals with an intellectual disability that prevents response to lifestyle questionnaires were excluded. Each case was evaluated with teachers and parents or legal guardians before exclusion. Participants who did not fully complete the KIDMED questionnaire (n = 484) or did not report their sex (n = 1) were excluded from the study. Lifestyle data of children/adolescents were self-reported online, with the assistance of trained researchers.

The PASOS study was conducted according to the guidelines of the Declaration of Helsinki and approved by the Ethics Committee of the Fundació Sant Joan de Déu, Barcelona, Spain. This committee does not give a number (please, see our paper on the protocol of the study [16]. A signed informed consent form was obtained from the parent or legal guardian of each participant. The trial was registered in 2019 at the International Standard Randomized Controlled Trial (ISRCT; https://doi.org/10.1186/ISRCTN34251612 (accessed on 19 July 2021) with the number 34251612.

### 2.3. Assessment of Mediterranean Diet Adherence

Participants were administered a validated 16-item KIDMED questionnaire [8] on their usual consumption, last year. The KIDMED index (Table 1), derived based on dichotomous response options (Yes/No), was created to estimate adherence to MedDiet in childhood, based on the principles that sustain MedDiet patterns. An affirmative answer to items denoting lower adherence was assigned a value of −1 (4 items) and those related to higher adherence were scored +1 (12 items). This index was used to classify subjects into three categories according to their adherence to MedDiet: ‘low’ = −4 to 3 points; ‘moderate’ = 4 to 7 points; and ‘optimal’ = 8 to 12 points.

### 2.4. Anthropometric Variables

Anthropometric variables for each child/adolescent were measured according to the World Health Organization (WHO) standardized protocol [17]. Body weight and height were measured with the child/adolescent in light clothing, without shoes. Measurements were performed using an electronic SECA 899 scale (recorded to the nearest 100 g), a portable SECA 217 stadiometer (to the nearest 1 mm), and a flexible, non-stretch SECA 201 metric tape (to the nearest 1 mm). Body mass index (BMI) was calculated using weight and height (kg/m^2^). BMI Z-score was calculated according to the WHO 2007 growth standards for 5–19-year-old children and adolescents [18], and the weight status category of each child/adolescent was determined by age and sex according to BMI Z-score cutoffs: severe obesity >3 standard deviation (SD); obesity >2SD & ≤3SD; overweight >1SD & ≤2SD; healthy weight ≥−2SD & ≤1SD; underweight <−2SD & ≥−3SD; and severe underweight <−3SD. Children/adolescents were categorized into three groups: underweight, healthy-weight, and overweight/obesity.

### 2.5. Physical Activity and Sedentary Behaviour

The validated PAU-7S, a seven-item self-reported questionnaire [19], was used to assess physical activity levels in children and adolescents. Six questions asked about physical activity frequency and duration in the previous week: (1) ‘How many days did you go for a walk?’, (2) ‘How many days did you participate in movement play during recess time?’, (3) ‘How many days did you participate in movement play during free time after school or during the weekend?’, (4) ‘How many days did you have physical exercise class at school?’, (5) ‘How many days did you play a team sport?’, and (6) ‘How many days did you play an individual sport?’. The response options about physical activity were shown in a table with a box for each day of the week, in which children could mark if they spent: (1) 0 min (no activity), (2) <30 min, (3) 30 min–1 h, (4) 1–1.5 h, or (5) >1.5 h. The final question asked about health status with ‘Yes/No’ option: ‘Were you sick last week or did anything prevent you from performing your usual physical activity?’ [15]. Only average weekly time (in minutes) spent in moderate-vigorous physical activity (MVPA) was calculated. Children/adolescents were categorized into two groups (<60 min/day; ≥60 min/day) based on compliance with MVPA daily recommendation [20].

Sedentary behavior was assessed by a validated screen-time sedentary behavior questionnaire [21], which asked about time spent (1) watching TV, (2) playing computer games, (3) playing console (video) games, and (4) using a mobile phone, separately for weekdays and weekends. Participants had to tick one of these categories: (1) 0 min, (2) 0–30 min, (3) 30–60 min, (4) 60–120 min, (5) 120–180 min, (6) 180–240 min, and (7) >240 min. The sedentary minutes per day was estimated as follows: category 1 (0 min), 2 (15 min), 3 (45 min), 4 (90 min), 5 (150 min), 6 (210 min), and 7 (241 min), respectively. Children and adolescents were categorized into two groups (<120 min/day; ≥120 min/day) based on compliance with screen time recommendations from the American Academy of Pediatrics [22].

### 2.6. Mothers/Females Legal Guardians’ Outcomes

Parental sociodemographic and lifestyle (physical activity and smoking) data were collected. Two sets of questionnaires were delivered to each participating child and adolescent, to be answered separately by up to two parents/legal guardians. Additional data on parental health habits were recorded via an online. Around 82% of mothers/female legal guardians responded as ‘adult 1’, which means that they were the person who spent more time with the child/adolescent. Therefore, only information from 3249 mothers/female legal guardians was used in this study.

The validated REGICOR (Registre Gironí del COR) short physical activity questionnaire was used [23], including standardized questions: weight (kg), height (cm), smoking habit (smoker; former smoker; never smoker), and educational level (primary/illiterate; secondary education; university education). Mothers/female legal guardians were classified into healthy-weight, BMI <25 kg/m^2^; overweight, 25 kg/m^2^ ≥ BMI < 30 kg/m^2^; and obesity, BMI ≥30 kg/m^2^. Mothers/female legal guardians were classified according to their physical activity practice into <300 METs·min/week and ≥300 METs·min/week [24,25]. Sedentary behavior was determined as total hours/day in front of screen, and mothers/female legal guardians were classified into <120 min/day and ≥120 min/day. Dietary habits of mothers/female legal guardians were assessed by the validated short diet quality screener (SDQS) questionnaire based on 18 foods/food groups consumption frequency [26]. For each mother/female legal guardian SDQS terciles were calculated.

### 2.7. Statistics

Analyses were performed with Statistical Package for Social Sciences version 25.0 (IBM SPSS Statistics for Windows, Chicago, IL, USA). Categorical variables were presented as frequencies and/or proportions and differences were calculated using a chi-squared test. Continuous variables were presented as mean and SD, and differences were tested using an unpaired Students’ t-test or ANOVA, using Bonferroni post hoc analysis. Equality of variances was assessed with Levene’s test. Logistic regression analysis with estimation of corresponding odds ratio (OR) and the 95% confidence interval (CI) were calculated to (1) examine the association between adherence to MedDiet (dependent variables), with low+moderate adherence as reference, and child/adolescent and their mother’s lifestyle characteristics (independent variables); (2) examine association between characteristics of the child/adolescent (independent variable) and their mother with children’s ‘yes’ answers in the KIDMED questionnaire (dependent variable). Reliability by Cronbach’s alpha was measured in all used scales, and values between 0.70–0.90 were obtained. Logistic regression analysis was adjusted for sex, age, and BMI, unless the variable was the one of interest, to control for potential confounders. Results were considered significant if *p*-value (2-tailed) <0.05.

## 3. Results

Table 2 shows the proportion of children that answered ‘yes’ or ‘no’ to each question of the KIDMED scale. The children showed a higher proportion of answers related to high adherence to Mediterranean diet than those related to low adherence.

Table 3 shows the adherence to MedDiet of the study population. Primary schoolers showed a higher adherence to MedDiet than their Secondary peers, with significant differences in the low, moderate, and optimal score prevalence. Optimal adherence to MedDiet was also observed in children/adolescents meeting daily recommended MVPA, as well as in those meeting daily recommended screen time on weekdays and weekends.

Table 4 shows the adherence to MedDiet of the study population according to the mother’s/female legal guardian’s sociodemographic and lifestyle characteristics. Educational level, SDQS score, BMI < 25 kg/m^2^, not smoking, meeting recommendation of <120 min/day of total screen time on weekdays and weekends, and meeting the physical activity recommendation of at least 300 METs·min/day were positive predictive factors of child/adolescent optimal adherence to MedDiet.

Table 5 shows the results of logistic regression for ‘yes’ answers in subcategories. Primary schooling appeared to be a positive predictive factor of consumption of dairy products, cereals, or grain products for breakfast; one fruit once a day or more than once a day; vegetables more than once a day; and fish more regularly, several times per week. Male sex appeared to be a positive predictive factor of consumption of dairy products for breakfast, pulses once a week, and pasta or rice almost every day. Meeting recommendations on physical activity and screen time was a positive predictive factor of consumption of dairy products (except for screen time) and cereals or grain products for breakfast, fruits once or more than once a day, raw or cooked vegetables once or more than once a day, pulses more than once a week, and fish and nuts more regularly several times per week. Overweight/obesity status was a positive predictive factor of consumption of raw or cooked vegetables more than once a day but also skipping breakfast.

Table 6 shows the results of logistic regression for ‘yes’ answers in mothers/female legal guardians’ subcategories. Higher educational level appeared to be a positive predictive factor of child/adolescent consumption of dairy products and cereals or grain products for breakfast, fruits more than once a day, vegetables once a day, two yogurts and/or some cheese daily, fish more regularly several times per week, and pulses once a week. The highest SDQS tercile appeared to be a positive predictive factor of child/adolescent consumption of dairy products and cereals or grain products for breakfast, fruits once a day or more than once a day, raw or cooked vegetables once a day, fish and nuts regularly, and pulses more than once per week. Meeting the recommended screen time on weekends appeared to be a positive predictive factor of child/adolescent consumption of fruits and vegetables once a day or more than once a day, and fish and nuts regularly; practicing physical activity more than 300 METs·min/day also appeared to be positive predictive factors of child/adolescent consumption of nuts. Non-smoking habit appeared to be a positive predictive factor of consumption of dairy products or cereals and grain products for breakfast, fruits once a day or more than once per day, raw or cooked vegetables once a day, and pasta or rice almost every day. Obesity appeared to be a negative predictive factor of child/adolescent consumption of dairy products for breakfast, fruits once a day or more than once a day, and nuts regularly, and it appeared to be a positive predictive factor of child/adolescent skipping breakfast, taking sweets and candies several times a day, and eating at fast-food restaurants more than once per week.

## 4. Discussion

The main findings of this study are that a low prevalence of optimal adherence to the MedDiet was found among current Spanish young people, and the main determinants for an optimal adherence to the MedDiet were reaching the recommendations on physical activity and screen time, highest maternal educational level, and healthier maternal lifestyles. These findings also confirm the need to improve adherence to MedDiet among the Spanish childhood population: only 45.5% of children (primary school students) and 34.8% of adolescents (secondary school students) showed optimal adherence to MedDiet, respectively.

Previous studies that aimed to explore adherence to the MedDiet among Spanish children and adolescents also obtained a low prevalence of optimal adherence [1,2,6,9,10,11,12,13,14]. Croatian 2–7-year-old children showed low KIDMED index score (49%), indicating a low MedDiet adherence, 37% had an average score, while only 14% had high MedDiet compliance [26]. In North Lebanon, adherence to the MedDiet was good amongst only 13.3% of adolescents [27]. However, there was significant variability in the samples analyzed, and direct comparisons could not be made between studies. Primary school students had a higher degree of adherence to MedDiet than secondary school students, which is in line with previous research [8,9,12,28]. This could be explained by adult supervision and control over children’s diets, which is associated with following a healthy dietary pattern. This influence is progressively lost as an individual grows up [12]. Optimal adherence to MedDiet was positively associated with meeting recommendations on physical activity and screen time. Optimal adherence to MedDiet was previously associated with physical activity practice [6,11,28,29], and it was inversely associated with screen time [6,11,28,29,30]. High levels of physical fitness were associated with high adherence to MedDiet [28,29,31,32]. Therefore, strategies are needed that target increasing physical activity and decreasing screen time, as these two habitual behaviors have demonstrated a relationship with MedDiet.

Recent studies found an inverse relationship between adherence to MedDiet and BMI [33,34,35], while others did not [6,13,36]. Negative correlations between mid-upper arm circumference and waist circumference and the consumption of a second daily serving of fruit, as well as a daily serving of vegetables, were previously found in other studies [26]. In the current study, consumption of dairy products and commercially baked goods or pastries for breakfast, as well as sweets or candies several times per day, were less frequent among overweight/obesity than healthy-weight students, while skipping breakfast and the consumption of raw or cooked vegetables more than once a day were more frequent among them. Children/adolescents may reduce the consumption of high energy density foods and increase the consumption of vegetables due to greater awareness of their overweight or obesity status [37]. The consumption of low nutritional density food, such as pastries and sweets, was also less usual among healthy-weight students than their underweight peers. Regarding the prevention of overweightness and obesity, it is extremely important to consider the synergistic value that behavior can exert, such as physical activity, screen time, and healthy diet.

Maternal maximum educational level and healthy lifestyle habits appeared to be positive predictive factors of children and adolescents’ optimal adherence to the MedDiet. Children and adolescents who had mothers with a higher educational level were more likely to present an optimal adherence to MedDiet [12]. Students who had mothers in the highest SDQS tercile were also more likely to present an optimal adherence to MedDiet. An association between parent diet quality and selected child dietary patterns was previously described. Parents with poor diet quality were more likely to have children that had a frequent consumption of snacks, which loaded positively for ice cream, non-dairy drinks, white bread, pasta and noodles, salty snacks, sweet baked items, lollies/sweets, sweet snacks, and sandwich spreads [38]. The prevalence of optimal adherence to MedDiet was also lower in children and adolescents whose mothers had a BMI of ≥30 kg/m^2^. Students who had mothers with a BMI ≥30 kg/m^2^ ate at fast-food restaurants more than once per week, took sweets and candies several times every day, and skipped breakfast; and they were less likely to consume dairy products for breakfast and fruits once or more than once a day, and consume nuts regularly. Fast-food purchases for family meals were previously associated with weight status among parents [39]. Children and adolescents whose mothers met physical activity and screen time recommendations were more likely to observe an optimal adherence to MedDiet. Lately, parental health status was positively associated with children’s eating habits and adherence to MedDiet [40]. Students who had mothers who never smoked or were former smokers presented an optimal adherence to MedDiet. Parental smoking habits were also associated with different dietary patterns among adolescents, regardless of whether adolescents themselves smoked [41].

In previous studies conducted in children and adolescents, maternal weight, smoking, and alcohol consumption were related to offspring obesity [42] and metabolic syndrome [43,44]. In the present study, maternal healthy lifestyle (characterized by a healthy body mass index, high-quality diet, regular exercise, low screen time, and no smoking) was associated with adherence to MedDiet in offspring, which emphasized the importance of maternal lifestyle, and suggested a combined effect of maternal and offspring lifestyle on child and adolescent diet. This study suggested that the development of mothers’ healthy lifestyles could provide further opportunity to improve offspring’s health, in addition to the improvement of child and adolescent lifestyle behavior. Intervening, by focusing on the lifestyle of mothers, but preferably on both members of the mother-offspring dyad, might be an effective strategy for increasing adherence to MedDiet.

## 5. Strengths and Limitations

The strengths of the current study were the relatively large and nationwide representative sample of Spanish children and adolescents, as well as the inclusion of relevant factors related to maternal history, which were not frequently included in previous research. These data are crucial for further investigations and description of nutrition transition phenomenon. The current study also has several limitations. First, the PASOS study is cross-sectional research and cannot establish causality for the significant associations studied. Second, KIDMED score was used, without integrating it with the food frequency questionnaire. However, the KIDMED test is the most used index of adherence to MedDiet in the pediatric literature, enabling comparison with the situation in other countries. Third, with the exception of children anthropometric data, other variables were obtained by means of questionnaires. Consequently, the current findings suffer from the inherent limitations of self-reported data, such as memory bias and influence of social desirability. Fourth, not all the questions of the surveys were responded to by all participants, as well as by all mothers/female legal guardians, which loses power by reducing the sample size. Since the sample size is indicated in each used variable, the results should be interpreted with caution. The knowledge, perceptions, and attitudes of participants towards healthy dietary patterns were factors not included in this study.

## 6. Conclusions

A low prevalence of optimal adherence to MedDiet was observed among Spanish children and adolescents. Meeting recommendations on physical activity, screen time, and having healthier and educated mothers were associated with optimal adherence to MedDiet.

## Figures and Tables

**Table 1:** Adherence to the Mediterranean Diet Questionnaire (KIDMED).

Q1. Skips breakfast (−)	Q9. Consumes raw or cooked vegetables more than 1/day (+)
Q2. Takes dairy product for breakfast (+)	Q10. Regular fish consumption (at least 2–3/week) (+)
Q3. Takes cereal or grains product for breakfast (+)	Q11. Goes >1/week fast food restaurant (−)
Q4. Takes pastries/commercially baked goods for breakfast (−)	Q12. Regular nut consumption (≥2–3/week) (+)
Q5. Takes a fruit or fruit juice daily (+)	Q13. Likes pulses and eats more than 1/week (+)
Q6. Takes a second serving of fruit daily (+)	Q14. Takes sweets and candies several times every day (−)
Q7. Consumes yogurts and/or 40 g cheese daily (+)	Q15. Consumes rice or pasta almost daily (≥5/week) (+)
Q8. Consumes raw or cooked vegetables daily (+)	Q16. Use of olive oil at home (+)

(+): healthy dietary habit; (−): unhealthy dietary habit.

**Table 2:** Proportion of children that answered ‘yes’ or ‘no’ to each question of the KIDMED scale.

Questionnaire KIDMED	Yes (n; %)	No (n; %)	*p* *
Q1. Skips breakfast (−)	451; 12.6%	3128; 87.4%	<0.001
Q2. Takes dairy product for breakfast (+)	2928; 81.8%	651; 18.2%	0.001
Q3. Takes cereal or grains product for breakfast (+)	22809; 63.7%	1299; 36.3%	<0.001
Q4. Takes pastries/commercially baked goods for breakfast (−)	1152; 32.2%	2427; 67.8%	<0.001
Q5. Takes a fruit or fruit juice daily (+)	2580; 72.1%	999; 27.9%	<0.001
Q6. Takes a second serving of fruit daily (+)	1815; 50.7%	1764; 49.3%	<0.001
Q7. Consumes yogurts and/or 40 g cheese daily (+)	2774; 77.5%	805; 22.5%	<0.001
Q8. Consumes raw or cooked vegetables daily (+)	2298; 64.2%	1281; 35.8%	0.005
Q9. Consumes raw or cooked vegetables more than 1/day (+)	1163; 32.5%	2416; 67.5%	<0.001
Q10. Regular fish consumption (at least 2–3/week) (+)	2215; 61.9%	1364; 38.1%	<0.001
Q11. Goes >1/week fast food restaurant (−)	820; 22.9%	2759; 77.1%	0.001
Q12. Regular nut consumption (≥2–3/week) (+)	1854; 51.8%	1725; 48.2%	0.001
Q13. Likes pulses and eats more than 1/week (+)	2495; 69.7%	1084; 30.3%	0.001
Q14. Takes sweets and candies several times every day (−)	780; 21.8%	2799; 78.2%	0.001
Q15. Consumes rice or pasta almost daily (≥5/week) (+)	1739; 48.6%	1840; 51.4%	0.027
Q16. Uses of olive oil at home (+)	3264; 91.2%	315; 8.8%	<0.001

(+): healthy dietary habit; (−): unhealthy dietary habit. * Differences Yes vs No by chi-square.

**Table 3:** Adherence to the MedDiet of the study population.

		KIDMED Score †		‡ KIDMED Category				OR (95%CI) of Optimal Adherence
	N	Mean ± SD	*p*	Low	Moderate	Optimal	*p*	
School grade								
Primary Education	1830	7.1 ± 2.5	<0.001	8.7 ^b^	45.8 ^c^	45.5 ^b,c^	<0.001	1.00 (ref.)
Secondary Education	1777	6.5 ± 2.5		11.7 ^b^	53.5 ^c^	34.8 ^b,c^		0.65 (0.56–0.75) ***
Sex								
Male	1742	6.8 ± 2.4	0.075	9.4	49.2	41.4	0.168	1.00 (ref.)
Female	1865	6.7 ± 2.5		10.9	50.0	39.1		0.91 (0.80–1.04)
BMI categories								
Underweight	290	6.4 ± 2.6	0.052	13.8	50.0	36.2	0.060	1.00 (ref.)
Healthy-weight	2037	6.8 ± 2.5		9.1	50.0	40.9		1.22 (0.95–1.58)
Overweight/obesity	1252	6.7 ± 2.5		11.2	48.6	40.2		1.13 (0.87–1.48)
MVPA ≥60 min/day								
No	746	5.7 ± 2.4	<0.001	19.0 ^a,b^	57.2 ^a,c^	23.7 ^b,c^	<0.001	1.00 (ref.)
Yes	2854	7.1 ± 2.4		7.9 ^a,b^	47.6 ^a,c^	44.5 ^b,c^		2.39 (1.97–2.89) ***
Total screen time weekends <120 min/day								
No	2858	6.6 ± 2.5	<0.001	11.3 ^a,b^	51.8 ^a,c^	36.9 ^b,c^	<0.001	1.00 (ref.)
Yes	738	7.6 ± 2.3		5.7 ^a,b^	41.3 ^a,c^	53.0 ^b,c^		1.76 (1.48–2.10) ***
Total screen time weekdays <120 min/day								
No	1973	6.2 ± 2.5	<0.001	13.9 ^a,b^	54.3 ^a,c^	31.8 ^b,c^	<0.001	1.00 (ref.)
Yes	1623	7.4 ± 2.3		5.6 ^a,b^	44.0 ^a,c^	50.4 ^b,c^		2.05 (1.77–2.38) ***

Abbreviations: OR, odds ratio; CI, confidence interval; BMI, body mass index; MVPA, moderate and vigorous physical activity; min, minutes; y-o, years old. Data are shown as † means ± standard deviations or ‡ percentages. Differences in means were tested by the Student’s *t*-test or ANOVA, and Bonferroni was used for post-hoc analysis. Differences in percentages were tested by chi-squared test. Different letters in rows shows statistically significant differences between groups (^a–c^) by the Bonferroni post hoc test (*p* < 0.05). Odds ratios (OR) with 95% confidence intervals (CI) were calculated by binary logistic regression analysis. The analysis was adjusted by sex, age, and BMI, unless the variable was the one of interest. Reference group = low + moderate; OR presented for optimal vs. low + moderate. Differences OR: *** *p* < 0.001.

**Table 4:** Adherence to the MedDiet of the study population among mother/female legal guardian characteristics.

		KIDMED Score †		‡ KIDMED Category				OR (95%CI) of Optimal Adherence
	n	Mean ± SD	*p*	Low	Moderate	Optimal	*p*	
Education level								
Primary/Illiterate	465	6.4 ± 2.4 ^b^	<0.001	11.8	55.1 ^c^	33.1^c^	<0.001	1.00 (ref.)
Secondary	1728	6.5 ± 2.5 ^c^		12.5 ^a,b^	50.4 ^a,c^	37.1 ^b,c^		1.18 (0.95–1.47)
University	1015	7.4 ± 2.4 ^b,c^		5.8 ^a,b^	45.3 ^a,c^	48.9 ^b,c^		1.87 (1.48–2.36) ***
SDQS score								
T1: 29.0–37.9	292	6.3 ± 2.6 ^a,b^	<0.001	14.0 ^a,b^	51.7 ^a,c^	34.2 ^b,c^	<0.001	1.00 (ref.)
T2: 38.0–39.9	220	7.1 ± 2.3 ^a^		5.9	49.5	44.5		1.55 (1.08–2.22) *
T3: 40.0–49.0	406	7.3 ± 2.4 ^b^		6.7 ^b^	43.1 ^c^	50.2 ^b,c^		1.95 (1.43–2.67) ***
BMI categories								
Healthy weight	1727	7.0 ± 2.5 ^b,c^	<0.001	8.4 ^a,b^	48.4 ^a,c^	43.2 ^b,c^		1.00 (ref.)
Overweight	867	6.6 ± 2.4 ^c^		11.9	49.7	38.4		0.82 (0.69–0.97) *
Obesity	395	6.3 ± 2.5 ^b^		14.4 ^b^	52.2 ^c^	33.4 ^b,c^		0.67 (0.52–0.85) **
Smoking habit								
Smoker	759	6.4 ± 2.5 ^a,b^	<0.001	13.0 ^b^	53.2 ^b^	33.7 ^b,c^	<0.001	1.00 (ref.)
Former smoker	786	6.9 ± 2.4 ^a^		8.7	48.5	42.9		1.52 (1.24–1.88) ***
Never smoked	1666	6.9 ± 2.5 ^b^		9.8	48.3	42.0		1.45 (1.21–1.74) ***
Screen time weekends <120 min/day								
No	1667	6.5 ± 2.5	<0.001	11.9 ^b^	52.8 ^c^	35.3 ^b,c^	<0.001	1.00 (ref.)
Yes	1326	7.1 ± 2.4		7.8 ^b^	46.0 ^c^	46.2 ^b,c^		1.53 (1.32–1.78) ***
Screen time weekdays <120 min/day								
No	780	6.4 ± 2.4	<0.001	12.9 ^b^	51.4	35.6 ^b^	0.001	1.00 (ref.)
Yes	2251	6.9 ± 2.5		9.1 ^b^	48.9	42.0 ^b^		1.24 (1.05–1.47) *
PA ≥300 METs*min/day ‡								
No	1142	6.7 ± 2.4	0.067	10.4	50.2	39.4	0.201	1.00 (ref.)
Yes	934	6.9 ± 2.6		10.4	46.5	43.1		1.20 (1.01–1.44) *

Abbreviations: OR, odds ratio; CI, confidence interval; BMI, body mass index; SDQS, short diet quality screener; T, tercile; PA, physical activity; METs, metabolic equivalents; min, minutes. Data are shown as † means ± standard deviations or ‡ percentages. Differences in means were tested by the Student’s *t*-test or ANOVA, and Bonferroni was used for post hoc analysis. Differences in percentages were tested by chi-squared test. Different letters in rows shows statistically significant differences between groups (^a–c^) by the Bonferroni post hoc test (*p* < 0.05). Odds ratio (OR) with 95% confidence intervals (CI) were calculated by binary logistic regression analysis. The analysis was adjusted by child/adolescent sex, age, and BMI. Reference group = low + moderate; OR presented for optimal vs. low + moderate. Differences OR: * *p* < 0.005; ** *p* < 0.01; *** *p* < 0.001.

**Table 5:** Odds ratios (and 95% confidence intervals) from logistic regression showing the association of child/adolescent characteristics with child/adolescent ‘yes’ answers in the KIDMED questionnaire.

		OR (95%CI) of ‘Yes’ Answers in Each Item of the KIDMED Questionnaire							
	n	Q1	Q2	Q3	Q4	Q5	Q6	Q7	Q8
School group									
Primary	1830	1.00 (ref.)	1.00 (ref.)	1.00 (ref.)	1.00 (ref.)	1.00 (ref.)	1.00 (ref.)	1.00 (ref.)	1.00 (ref.)
Secondary	1777	2.24 (1.79–2.80) ***	0.81 (0.67–0.97) *	0.78 (0.68–0.91) **	1.19 (1.02–1.39) *	0.68 (0.58–0.80) ***	0.51 (0.44–0.58) ***	1.38 (1.16–1.63) ***	0.89 (0.77–1.04)
Sex									
Male	1742	1.00 (ref.)	1.00 (ref.)	1.00 (ref.)	1.00 (ref.)	1.00 (ref.)	1.00 (ref.)	1.00 (ref.)	1.00 (ref.)
Female	1865	1.67 (1.36–2.06) ***	0.76 (0.64–0.90) **	0.96 (0.83–1.10)	1.00 (0.87–1.15)	1.05 (0.91–1.22)	1.09 (0.95–1.25)	1.03 (0.88–1.21)	1.13 (0.99–1.30)
BMI classification									
Underweight	290	1.00 (ref.)	1.00 (ref.)	1.00 (ref.)	1.00 (ref.)	1.00 (ref.)	1.00 (ref.)	1.00 (ref.)	1.00 (ref.)
Healthy weight	2037	0.98 (0.66–1.45)	1.01 (0.72–1.41)	1.12 (0.87–1.45)	0.74 (0.57–0.95) *	1.15 (0.88–1.50)	1.24 (0.96–1.59)	1.01 (0.75–1.37)	1.07 (0.83–1.38)
Overweight/obesity	1252	1.53 (1.02–2.29) *	0.66 (0.47–0.93) *	0.89 (0.69–1.17)	0.52 (0.40–0.68) ***	1.14 (0.86–1.51)	1.17 (0.90–1.52)	0.89 (0.65–1.21)	1.07 (0.82–1.39)
MVPA ≥ 60 min/day									
No	746	1.00 (ref.)	1.00 (ref.)	1.00 (ref.)	1.00 (ref.)	1.00 (ref.)	1.00 (ref.)	1.00 (ref.)	1.00 (ref.)
Yes	2854	0.91 (0.72–1.16)	1.36 (1.11–1.67) **	1.62 (1.36–1.92) ***	0.80 (0.67–0.96) *	1.72 (1.44–2.06) ***	2.22 (1.86–2.65) ***	1.20 (0.98–1.47)	1.65 (1.39–1.97) ***
Total screen time <120 min/day									
No	2430	1.00 (ref.)	1.00 (ref.)	1.00 (ref.)	1.00 (ref.)	1.00 (ref.)	1.00 (ref.)	1.00 (ref.)	1.00 (ref.)
Yes	1149	0.64 (0.48–0.84) **	1.23 (1.00–1.51)	1.29 (1.09–1.52) **	0.64 (0.54–0.77) ***	1.58 (1.32–1.90) ***	1.76 (1.50–2.06) ***	0.91 (0.76–1.09)	1.71 (1.44–2.02) ***
	n	Q9	Q10	Q11	Q12	Q13	Q14	Q15	Q16
School group									
Primary	1830	1.00 (ref.)	1.00 (ref.)	1.00 (ref.)	1.00 (ref.)	1.00 (ref.)	1.00 (ref.)	1.00 (ref.)	1.00 (ref.)
Secondary	1777	0.62 (0.53–0.72) ***	0.75 (0.65–0.87) ***	1.26 (1.07–1.49) **	0.91 (0.79–1.05)	1.05 (0.90–1.22)	1.56 (1.32–1.86) ***	1.26 (1.10–1.45) **	2.85 (2.17-3.74) ***
Sex									
Male	1742	1.00 (ref.)	1.00 (ref.)	1.00 (ref.)	1.00 (ref.)	1.00 (ref.)	1.00 (ref.)	1.00 (ref.)	1.00 (ref.)
Female	1865	1.04 (0.91–1.20)	0.89 (0.77–1.01)	1.00 (0.86–1.17)	0.96 (0.84–1.09)	0.85 (0.74–0.98) *	1.22 (1.04–1.43) *	0.86 (0.75–1.00) *	1.53 (1.21–1.94) ***
BMI classification									
Underweight	290	1.00 (ref.)	1.00 (ref.)	1.00 (ref.)	1.00 (ref.)	1.00 (ref.)	1.00 (ref.)	1.00 (ref.)	1.00 (ref.)
Healthy weight	2037	1.17 (0.89–1.54)	1.25 (0.97–1.61)	0.82 (0.62–1.08)	0.99 (0.77–1.27)	1.12 (0.86–1.46)	0.61 (0.47–0.80) ***	0.84 (0.65–1.07)	0.81 (0.49–1.34)
Overweight/obesity	1252	1.38 (1.04–1.83) *	1.13 (0.87–0.47)	0.78 (0.58–1.04)	0.80 (0.62–1.04)	1.16 (0.88–1.53)	0.48 (0.36–0.64) ***	0.81 (0.62–1.04)	0.67 (0.40–1.11)
MVPA ≥ 60 min/day									
No	746	1.00 (ref.)	1.00 (ref.)	1.00 (ref.)	1.00 (ref.)	1.00 (ref.)	1.00 (ref.)	1.00 (ref.)	1.00 (ref.)
Yes	2854	1.84 (1.51–2.24) ***	1.76 (1.48–2.09) ***	0.96 (0.79–1.17)	1.80 (1.52–2.14) ***	1.60 (1.34–1.91) ***	0.63 (0.52–0.77) ***	1.07 (0.90–1.26)	1.24 (0.90–1.70)
Total screen time <120 min/day									
No	2430	1.00 (ref.)	1.00 (ref.)	1.00 (ref.)	1.00 (ref.)	1.00 (ref.)	1.00 (ref.)	1.00 (ref.)	1.00 (ref.)
Yes	1149	1.37(1.17–1.62) ***	1.62 (1.37–1.91) ***	0.51 (0.42–0.62) ***	1.40 (1.20–1.64) ***	1.59 (1.34–1.89) ***	0.46 (0.37–0.56) ***	0.94 (0.80–1.10)	0.97 (0.75–1.26)

Q1–Q16: items are described in Table 1. Abbreviations: OR, odds ratio; CI, confidence interval; Q, question; MVPA, moderate and vigorous physical activity; min, minutes; y-o, years old. Odds ratio (OR) with 95% confidence intervals (CI) were calculated by binary logistic regression analysis with ‘no’ answers as reference category in dependent variable. The analysis was adjusted by sex, age, and BMI, unless the variable was the one of interest. Differences OR: * *p* < 0.005; ** *p* < 0.01; *** *p* < 0.001.

**Table 6:** Odds ratios (and 95% confidence intervals) from logistic regression showing the association of child/adolescent characteristics with child/adolescent ‘yes’ answers in the KIDMED questionnaire.

		OR (95%CI) of ‘Yes’ Answers in Each Item of the KIDMED Questionnaire							
	n	Q1	Q2	Q3	Q4	Q5	Q6	Q7	Q8
Maximum education level									
Primary/Illiterate	465	1.00 (ref.)	1.00 (ref.)	1.00 (ref.)	1.00 (ref.)	1.00 (ref.)	1.00 (ref.)	1.00 (ref.)	1.00 (ref.)
Secondary	1728	0.87 (0.65–1.17)	0.98 (0.76–1.27)	1.02 (0.83–1.27)	1.01 (0.81–1.26)	0.83 (0.66–1.04)	0.89 (0.72–1.10)	1.29 (1.02–1.63) *	1.07 (0.87–1.32)
University	1015	0.65 (0.46–0.91) *	1.52 (1.14–2.04) **	1.29 (1.03–1.63) *	1.01 (0.79–1.28)	1.23 (0.95–1.59)	1.47 (1.18–1.84) **	1.51 (1.16–1.96) **	1.47 (1.17–1.86) **
SDQS score									
T1: 29.0–37.9	292	1.00 (ref.)	1.00 (ref.)	1.00 (ref.)	1.00 (ref.)	1.00 (ref.)	1.00 (ref.)	1.00 (ref.)	1.00 (ref.)
T2: 38.0–39.9	220	0.68 (0.36–1.30)	1.61 (0.98–2.64)	1.26 (0.87–1.82)	0.89 (0.61–1.29)	1.34 (0.91–1.98)	1.33 (0.93–1.90)	1.47 (0.94–2.31)	1.39 (0.96–2.01)
T3: 40.0–49.0	406	0.84 (0.50–1.41)	1.80 (1.18–2.74) **	1.41 (1.03–1.94) *	1.01 (0.73–1.39)	1.43 (1.02–1.99) *	1.72 (1.27–2.35) **	1.25 (0.86–1.81)	1.45 (1.05–1.98) *
BMI categories									
Healthy weight	1727	1.00 (ref.)	1.00 (ref.)	1.00 (ref.)	1.00 (ref.)	1.00 (ref.)	1.00 (ref.)	1.00 (ref.)	1.00 (ref.)
Overweight	867	1.18 (0.90–1.54)	0.94 (0.75–1.16)	0.86 (0.72–1.02)	1.22 (1.02–1.45) *	0.94 (0.78–1.13)	0.91 (0.77–1.08)	1.07 (0.88–1.31)	0.89 (0.75–1.05)
Obesity	395	1.58 (1.14–2.19) **	0.67 (0.51–0.88) **	0.88 (0.70–1.12)	1.17 (0.91–1.49)	0.70 (0.55–0.90) **	0.68 (0.54–0.86) **	1.02 (0.77–1.34)	0.84 (0.67–1.06)
Smoking habit									
Smoker	759	1.00 (ref.)	1.00 (ref.)	1.00 (ref.)	1.00 (ref.)	1.00 (ref.)	1.00 (ref.)	1.00 (ref.)	1.00 (ref.)
Former smoker	786	0.71 (0.52–0.96) *	1.33 (1.03–1.71) *	1.21 (0.98–1.49)	0.86 (0.70–1.06)	1.13 (0.91–1.41)	1.39 (1.13–1.71) **	1.23 (0.97–1.57)	1.17 (0.95–1.44)
Never smoked	1666	0.79 (0.61–1.03)	1.40 (1.13–1.73) **	1.21 (1.01–1.45) *	0.75 (0.62–0.90) **	1.21 (1.00–1.47) *	1.20 (1.01–1.43) *	1.22 (0.99–1.49)	1.22 (1.02–1.46) *
Screen time weekends <120 min/day									
No	1667	1.00 (ref.)	1.00 (ref.)	1.00 (ref.)	1.00 (ref.)	1.00 (ref.)	1.00 (ref.)	1.00 (ref.)	1.00 (ref.)
Yes	1326	0.87 (0.69–1.9)	1.01 (0.83–1.21)	1.23 (1.06–1.43) **	0.84 (0.72–0.98) *	1.27 (1.08–1.49) **	1.45 (1.25–1.69) ***	0.85 (0.71–1.01)	1.26 (1.09–1.47) **
Screen time weekdays <120 min/day									
No	780	1.00 (ref.)	1.00 (ref.)	1.00 (ref.)	1.00 (ref.)	1.00 (ref.)	1.00 (ref.)	1.00 (ref.)	1.00 (ref.)
Yes	2251	0.83 (0.65–1.06)	1.16 (0.94–1.43)	1.16 (0.98–1.38)	0.85 (0.71–1.01)	1.27 (1.06–1.52) **	1.35 (1.14–1.59) ***	0.95 (0.78–1.16)	1.22 (1.03–1.44) *
PA ≥300 METs * min/day ‡									
No	1142	1.00 (ref.)	1.00 (ref.)	1.00 (ref.)	1.00 (ref.)	1.00 (ref.)	1.00 (ref.)	1.00 (ref.)	1.00 (ref.)
Yes	934	0.90 (0.68–1.19)	0.87 (0.69–1.10)	1.00 (0.83–1.20)	0.95 (0.78–1.14)	1.11 (0.91–0.34)	1.19 (1.00–1.43)	1.11 (0.90–1.37)	1.07 (0.89–1.28)
	n	Q9	Q10	Q11	Q12	Q13	Q14	Q15	Q16
Maximum education level									
Primary/Illiterate	465	1.00 (ref.)	1.00 (ref.)	1.00 (ref.)	1.00 (ref.)	1.00 (ref.)	1.00 (ref.)	1.00 (ref.)	1.00 (ref.)
Secondary	1728	1.11(0.88–1.39)	0.92 (0.74–1.13)	0.85 (0.67–1.07)	0.98 (0.80–1.21)	1.16 (0.93–1.45)	0.84 (0.66–1.06)	1.01 (0.82–1.24)	1.09 (0.76–1.56)
University	1015	1.21(0.95–1.54)	1.60 (1.27–2.02) ***	0.51 (0.39–0.67) ***	1.20 (0.96–1.50)	1.37 (1.08–1.74) **	0.49 (0.37–0.64) ***	0.83 (0.67–1.04)	1.48 (0.99–2.22)
SDQS score									
T1: 29.0–37.9	292	1.00 (ref.)	1.00 (ref.)	1.00 (ref.)	1.00 (ref.)	1.00 (ref.)	1.00 (ref.)	1.00 (ref.)	1.00 (ref.)
T2: 38.0–39.9	220	0.99 (0.67–1.47)	1.43 (0.99–2.06)	0.68 (0.43–1.09)	1.21(0.85–1.73)	1.46 (0.99–2.16)	0.92 (0.58–1.45)	1.20 (0.84–1.70)	1.32 (0.71–2.43)
T3: 40.0–49.0	406	1.38 (0.99–1.92)	1.65 (1.20–2.27) **	0.99 (0.69–1.44)	1.63 (1.20–2.21) **	1.49 (1.06–2.08) *	0.83 (0.56–1.24)	1.10 (0.81–1.49)	1.52 (0.89–2.60)
BMI categories									
Healthy weight	1727	1.00 (ref.)	1.00 (ref.)	1.00 (ref.)	1.00 (ref.)	1.00 (ref.)	1.00 (ref.)	1.00 (ref.)	1.00 (ref.)
Overweight	867	1.11 (0.93–1.32)	0.78 (0.66–0.93) **	1.56 (1.29–1.89) ***	0.83 (0.70–0.98) *	0.93 (0.78–1.11)	1.32 (1.08–1.61) **	1.21 (1.02–1.42) *	0.75 (0.56–1.02)
Obesity	395	0.99 (0.77–1.26)	0.81 (0.64–1.02)	1.44 (1.11–1.88) **	0.77 (0.61–0.96) *	0.98 (0.77–1.26)	1.60 (1.23–2.10) **	1.19 (0.95–1.50)	0.77 (0.51–1.16)
Smoking habit									
Smoker	759	1.00 (ref.)	1.00 (ref.)	1.00 (ref.)	1.00 (ref.)	1.00 (ref.)	1.00 (ref.)	1.00 (ref.)	1.00 (ref.)
Former smoker	786	1.04 (0.83–1.29)	1.25 (1.01–1.53) *	0.71 (0.56–0.90) **	1.20 (0.98–1.47)	1.03 (0.82–1.28)	0.58 (0.46–0.74) ***	1.01 (0.83–1.24)	1.21 (0.82–1.80)
Never smoked	1666	1.19 (0.99–1.44)	1.10 (0.92–1.31)	0.87 (0.72–1.07)	1.01 (0.85–1.20)	0.87 (0.72–1.05)	0.68 (0.55–0.83) ***	1.27 (1.07–1.51) **	0.81 (0.59–1.11)
Screen time weekends <120 min/day									
No	1667	1.00 (ref.)	1.00 (ref.)	1.00 (ref.)	1.00 (ref.)	1.00 (ref.)	1.00 (ref.)	1.00 (ref.)	1.00 (ref.)
Yes	1326	1.44 (1.23–1.69) ***	1.28 (1.10–1.49) **	0.61 (0.51–0.73) ***	1.25 (1.08–1.45) **	1.06 (0.91–1.24)	0.80 (0.67–0.96) *	1.05 (0.91–1.22)	1.09 (0.83–1.42)
Screen time weekdays <120 min/day									
No	780	1.00 (ref.)	1.00 (ref.)	1.00 (ref.)	1.00 (ref.)	1.00 (ref.)	1.00 (ref.)	1.00 (ref.)	1.00 (ref.)
Yes	2251	1.17 (0.98–1.40)	1.17 (0.99–1.39)	0.66 (0.55–0.80) ***	1.17 (0.99–1.37)	0.84 (0.70–1.01)	0.83 (0.68–1.01)	0.86 (0.73–1.01)	1.06 (0.78–1.44)
PA ≥300 METs * min/day ‡									
No	1142	1.00 (ref.)	1.00 (ref.)	1.00 (ref.)	1.00 (ref.)	1.00 (ref.)	1.00 (ref.)	1.00 (ref.)	1.00 (ref.)
Yes	934	1.02 (0.85–1.23)	1.08 (0.90–1.29)	0.81 (0.66–1.00)	1.29 (1.08–1.54) **	0.96 (0.80–1.17)	0.83 (0.67–1.03)	0.93 (0.78–1.11)	1.05 (0.76–1.45)

Q1–Q16: items are described in Table 1. Abbreviations: OR, odds ratio; CI, confidence interval; SDQS, short diet quality screener; Q, question; min, minutes; y-o, years old. Odds ratio (OR) with 95% confidence intervals (CI) were calculated by binary logistic regression analysis with ‘no’ answers as reference category in dependent variable. The analysis was adjusted by sex, age, and BMI, unless the variable was the one of interest. Differences OR: * *p* < 0.005; ** *p* < 0.01; *** *p* < 0.001.

## Data Availability

There are restrictions on the availability of data for this trial, due to the signed consent agreements and around data sharing, which only allow access to external researchers for studies following the project purposes. Requestors wishing to access the trial data used in this study can make a request to pep.tur@uib.es.
